# Correlations between the anti-corrosion properties and the photocatalytic behavior of epoxy coatings incorporating modified graphene oxide deposited on a zinc substrate†

**DOI:** 10.1039/d4ra00413b

**Published:** 2024-04-03

**Authors:** Tamara-Rita Ovari, Boglárka Trufán, Gabriel Katona, Gabriella Szabó, Liana Maria Muresan

**Affiliations:** a Department of Chemical Engineering Romania; b Department of Chemistry and Chemical Engineering, Hungarian Line, Babes-Bolyai University, Faculty of Chemistry and Chemical Engineering 11, Arany J. St 400028 Cluj-Napoca Romania gabriella.szabo@ubbcluj.ro

## Abstract

This research aimed to create a substrate-coating system based on zinc and an epoxy resin incorporating modified graphene oxide, which possesses two key characteristics: effective resistance against corrosion and the ability to harness photocatalytic properties. Furthermore, correlations between the anti-corrosion properties and the photocatalytic behaviour of the coatings were made. Thin epoxy (EP) layers embedding 0.1 wt% graphene oxide (GO), reduced graphene oxide (rGO), and modified graphene oxide with (3-aminopropyl)-triethoxysilane (APTES) or poly(amidoamine) (PAMAM) dendrimer were applied on a zinc (Zn) substrate using the dip-coating method. Anti-corrosion properties of coated Zn samples were investigated through electrochemical impedance spectroscopy (EIS) measurements. They showed that the corrosion protection effect is more prominent for EP containing functionalized GO, the highest in the case of GO-PAMAM. The results of the EIS measurements indicated also that the corrosion protection provided by EP-rGO is smaller than that of EP. The photocatalytic properties of the coatings were studied by exposure of the samples to Methylene Blue (MB) solution followed by monitoring the model dye degradation through UV-Vis measurements. To determine the changes in the anti-corrosion properties due to photocatalysis, the coated Zn samples were put through additional EIS measurements. The same coatings applied to a glass substrate lacked photocatalytic properties, indicating that the Zn substrate is accountable for the degradation of MB. Furthermore, the incorporation of GO or functionalized GO into the coating amplifies this effect. From EIS spectra, it was determined that the protective properties loss observed after 3 days is due to coating delamination during exposure to MB solution, the EP-GO-APTES retaining the best adhesion of the coating, 98% remaining on Zn after a cross-hatch test. The corrosion measurements were complemented by examining the morphology and structure of the coatings and the modified GO particles. All things considered, the Zn/EP-GO-APTES system shows the best ability to break down organic pollutants, keeping a good anti-corrosive property and adhesion.

## Abstract 2

Modified GO NPs were added to EP coatings on Zn to study their electrochemical, photocatalytic, and morphological properties. The Zn/EP-GO-APTES system showed the best organic pollutant degradation, keeping good corrosion resistance and adhesion.

## Graphical abstract

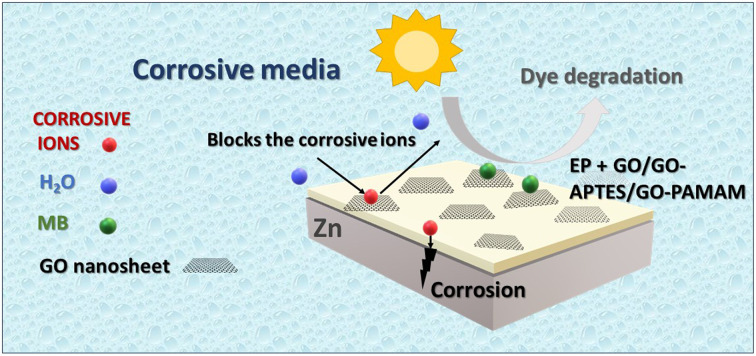

## 1 Introduction

One of the efficient ways to avoid the oxidation of metals due to aggressive environmental effects is through coatings application on their surface. In the past few decades, a continuously increased interest has been observed in the preparation of sophisticated eco-friendly anti-corrosion coatings to substitute highly toxic chromate treatments^1,2^ with intensified interest for silica,^3–7^ titania,^8,9^ biopolymers^10–12^ and organic-based protective layers.^13^ Among these, epoxy is a widespread polymer matrix for the protection of metal surfaces, due to its good physical barrier effect.^14,15^ Although it has high corrosion resistance, good adhesion, and thermal resistance, its application is overshadowed by poor mechanical properties, like high rigidity, brittleness that is prone to cracks, and moisture absorption. The corroding agents can penetrate through the pores and defects of the coating causing its long-time protection failure. To improve the aforementioned weakness, there were reports of incorporating various types of fillers into the matrix.^16,17^ The primary objectives involve strengthening the mechanical properties of the epoxy matrix and impeding the diffusion of the electrolyte.

Mechanical properties like fracture toughness, strength, and tensile modulus of the composites were significantly enhanced as a result of bonding between the epoxy matrix and silica-modified graphene–oxide (GO) hybrid,^18,19^ or diglycidyl ether of bisphenol-A functionalized GO,^20^ or hyperbranched polymers.^21^ Improvement of the coatings' adherence can be achieved by pretreatment of the metal substrates. On aluminium alloys, adherence is enhanced by silane film deposition,^22^ on mild steel, with silane^23^ or using functionalized GO,^24–27^ or on copper, by coating with a graphene (G) reinforced composite.^24^

Diminished permeability can be achieved by the addition of two-dimensional nanofillers^28^ like hexagonal boron nitride, G,^29^ or GO^30^ that provide tortuosity to the corroding agents' pathway. Although their high specific surface area is responsible for the barrier properties, simultaneously, the high surface energy and the interaction between the GO sheets lead to the formation of large-size aggregates. Naturally, agglomeration of the GO sheets can hinder their interaction ability with the epoxy, reduce the dispersity degree, consequently increase the brittleness, and lead to the formation of new electrolyte pathways. A possible way to avoid this shortcoming is through the functionalization of the nanoparticles. GO is suitable for surface modification through its several oxygen-containing functional groups (–OH, epoxide group on the basal plane, –COOH on the edge) which can act as reactive sites toward coupling with different agents and to form bonds. The nanoparticles,^31^ nanohybrids,^32^ and polymer chains^33^ grafted on the surface of the GO behave as spacers and can generate a loose structure of the sheets, finally preventing their assembly. Moreover, the interaction between the nanofiller and the polymer matrix can be also improved by functionalization. Although there were reported several possibilities for GO functionalization, the effectiveness of amine-terminated compounds was put in evidence. Among many GO functionalization ways reported by B. Ramezanzadeh *et al.*, it is worth highlighting some, such as those with *p*-phenylenediamine,^26^ (3-aminopropyl)-triethoxysilane (APTES)^27^ or with poly (amidoamine) (PAMAM) dendrimer.^34^ The APTES form two types of covalent bonds; its amino groups react with epoxide groups on the basal plane of the GO, while the hydrolysis-generated hydroxyl groups react with the carboxyl groups from the edge of GO.^27^ Even if APTES functionalized GO presented better compatibility with epoxy than the unmodified GO, the enhanced adhesion and corrosion resistance of the composite coating depend very much on the molar ratio of nanofiller/epoxy.^35^ By the functionalization of GO with PAMAM, covalent bonds are generated between GO's functional groups (mostly on the edge) and the dendrimer primer's amino groups.^34^ These groups of the GO-PAMAM interact either with the epoxide groups of the resin, generating an increased cross-linking of the composite, and thus enhancing its barrier properties, or with the metal oxide from the substrate's surface leading to a better adhesion. The surface properties of the prepared coatings vary depending on the substrate, leading to different results when testing the same coating on different surfaces. To our knowledge, the aforementioned coatings were not studied on Zn substrates.

Looking at it from a different perspective, GO and GO functionalized in various ways present photocatalytic activity too. This property was put in evidence by dyes decomposition, like methylene blue (MB), azo-dye plasmocorinth B (PB), crystal violet (CV), or rhodamine B (RB). It has to be mentioned, that in the majority of the cases, the utilization of GO was as composite nanoparticles,^36,37^ as GO-coated nanorods,^38^ as composite quantum dots,^39^ or as electrospun nanofibers.^40^ The main utilization of them was for pollutants, organic contamination removal from wastewaters, introduced as freely suspended particles or grafted on membranes. In some cases, was reported the introduction of them in TiO_2_ and ZnO thin coatings.^41–43^ To the best of our knowledge, the photocatalytic activity of the modified GO/epoxy coatings has not been reported thus far.

As eco-friendly technology continues to advance, the exploration of new materials with added functionalities has emerged as a key focus of research. Self-cleaning materials represent one group of intelligent systems.^44^ The self-cleaning property can be activated through photocatalysis and unique wettability, allowing it to autonomously remove contaminants adhered to its surface in diverse manners. Photocatalysis can provide an antibacterial, antimicrobial performance to the surface too.

In this context, this work aimed to investigate the possibility of corrosion-resistant coatings on zinc to act also as an environmentally friendly system, involving the degradation of pollutants, due to the embedded particles' photocatalytic properties. Moreover, possible correlations between the photocatalytic activity of the system composed of GO-APTES and GO-PAMAM-modified epoxy coatings on Zn substrate, and their anti-corrosion properties were investigated. For this reason, the coatings applied on Zn wafers were put through to electrochemical measurements before and after their submission to visible light, tracking their photocatalytic activity by decomposition of MB model substance. The MB decomposition was observed spectrophotometrically and from the obtained data the rate of reaction was determined. The experimental data delivered by Electrochemical Impedance Spectroscopy (EIS) measurements gave useful information regarding the mechanism of corrosion, delamination, and the integrity of the coatings. Also, it was important to monitor the changes caused by photocatalytic activity in the coatings' barrier properties and to understand the effect of different types of nanofillers on the composite coatings' anti-corrosion resistance.

## 2 Experimental

### 2.1. Materials

The epoxy resin was purchased from MAPEI Romania and the zinc plates (99% purity) with reduced amounts of Ti and Cu, from Altdepozit, Romania. The methylene blue (MB) was sourced from Merck (Germany), the isopropanol from CHEMICAL Company (Iasi, Romania), KCl from Primexchim (Bucharest, Romania), while the HCl (35–38% concentration) and the NaCl used for the preparation of the 3 wt% electrolyte was purchased from Chempur (Karlsruhe, Germany). The zinc samples (procured from Altdepozit, with 99% purity and minimal levels of Ti and Cu), measuring 7 × 2 cm, were polished using emery paper (grade 800, 2000, 5000). The surface area was limited to 2 cm^2^ by applying a transparent lacquer, defining the active surface area of the sample for electrochemical measurements. The Menzel-Gläser glass microscope slides were purchased from Thermo Scientific.

The reduced graphene oxide (rGO) and graphene oxide (GO) were procured from the National Institute for Research and Development of Isotopic Molecular Technologies, Cluj-Napoca. For modifying the GO, we utilized 3-aminopropyltriethoxysilane (APTES, 98%, Alfa Aesar), sodium hydroxide (NaOH, 97%, Lachema), and glacial acetic acid (CH_3_COOH, 99.5%, Chemical Company). Modification with poly(amidoamine) 2nd generation dendrimer (PAMAM G2.0, 38%, Dendritech) involved dimethylformamide (DMF, 99.5%, Thermo Scientific) and thionyl chloride (SOCl_2_, 99%, Sigma-Aldrich). All chemicals were of analytical grade and used without any purification step.

### 2.2. Functionalization of the GO

The functionalization of GO was achieved *via* a hydrolysis-condensation process using the organosilane APTES. Initially, the GO (0.031 g) was sonicated for 1 h in 12.5 mL of an aqueous solution. Another solution was prepared containing 1.32 mL of APTES, 0.3 mL of water, and 29.65 mL of ethanol. Upon mixing the two solutions, the pH was adjusted to 4.5 by adding acetic acid while stirring continuously for 2 h. Subsequently, the pH was raised to 11 using sodium hydroxide, and the mixture was stirred for an additional hour at room temperature. After filtration to remove the supernatant, the GO-APTES residue was washed with deionized water and ethanol (30 : 20 w/w).^7,45^

To modify the GO with PAMAM G2.0, activation of the carboxyl groups on the GO surface was necessary to facilitate nucleophilic substitution. This process involved the use of thionyl chloride (SOCl_2_) and dimethylformamide (DMF) in the following ratio: 0.15 g GO, 37.5 mL SOCl_2_, and 0.6 mL DMF, stirred at 70 °C for 24 h. Subsequently, the mixture was filtered to remove excess SOCl_2_, leaving behind GO-COCl. This compound was then dispersed in 15 mL of ethanol along with the PAMAM dendrimer. Excess EtOH was removed through filtration and subsequent rinsing with water. The resulting powder was dried at 60 °C to obtain GO-PAMAM.^7,46^

### 2.3. Preparation of the epoxy precursors

The epoxy precursor (bisphenol A epoxy resin) consisted of two elements: component A, which was the epoxy, and component B, which was the hardener composed of a blend of amine-containing monomers and oligomers. Mechanical stirring was used to combine them in a ratio of 3 : 1. The EP transformed into a gel-like state after 90 minutes, during which it lost its workability. To attain complete curing, the samples were allowed to sit at room temperature (25 °C) for at least 4 days. When the epoxy included various graphene oxides, the graphene nanofillers were added to the hardener and ultrasonicated (frequencies >20 kHz) for 10 minutes prior the mixing with EP resin to disperse the nanoparticles uniformly by breaking down aggregates. Nanoparticles can be better dispersed in the hardener due to its lower viscosity compared to the epoxy resin. This facilitates more thorough mixing and ensures uniform dispersion of nanoparticles throughout the composite material.^47^

### 2.4. Samples preparation

**Fig. 1:**
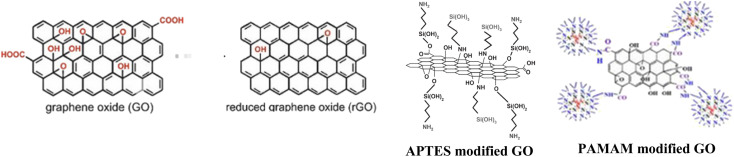
Graphic illustration of the used graphene oxides.

The initial phase of sample preparation involves the pre-treating of the substrates. The zinc plates were polished with emery paper (P800, P2000, P5000). Further, the elimination of particle residue was achieved through a process involving washing and ultrasonication in isopropanol for 2 minutes, followed by treatment with 0.1 M aqueous HCl solution to remove any remaining oxidization. Finally, the zinc plates were subjected again to a cleaning process involving the use of a cotton swab soaked in isopropanol, followed by a subsequent washing with isopropanol. The graphene nanofillers were added to the hardener and ultrasonicated prior the mixing with EP resin. The prepared epoxy resin contained the following nanoparticles in a concentration of 0.1 wt%:^47^ GO, reduced GO, GO-APTES and GO-PAMAM (Fig. 1). Afterwards, the prepared epoxy resin precursors were used to coat the samples at a rate of 5 cm min^−1^ by dip coating technique (thickness of the layers: ∼24–27 µm).

### 2.5. Fourier transformed-infrared spectroscopy (FT-IR) measurements

To emphasize the formation of bonds between modified GO and EP, Fourier-transform infrared (FT-IR) spectra were recorded. JASCO FT/IR-6800 spectrometer was used within the range of 500–4000 cm^−1^. The samples were prepared by mechanically removing a part of the cured epoxy with or without the graphene nanofillers and simply adding a small quantity of the solid samples into the spectrometer.

### 2.6. Coating thickness

The thickness of the coating was measured using a Trotec instrument called BB25, which utilizes the eddy current method to determine layer thicknesses on surfaces. The instrument has an accuracy of approximately ±1%. Three measurements were taken for each sample, and an average value was calculated.

### 2.7. Electrochemical investigation

The corrosive–resistant characteristics of the coatings were investigated using a PARSTAT-2273 single-channel potentiostat (Princeton Applied Research, Oak Ridge, TN, USA) within a three-electrode cell configuration. In this setup, the working electrode was the Zn substrate coated with the material being studied, possessing an active area of 2 cm^2^. The reference electrode used was Ag/AgCl with saturated KCl, and a Pt wire served as the counter electrode. The corrosive electrolyte solution employed was a 3 wt% NaCl solution. Open circuit potentials (OCPs) were measured for 30 minutes after immersion, which allowed for the attainment of stable OCP values across all systems. Additionally, impedance spectra were recorded at OCP using a 10 mV potential perturbation within the frequency range of 0.01 Hz to 10 kHz. The impedance measurements were repeated after 3 days of exposure in 10^−3^ M MB solution. The electrical circuit was fitted using the ZsimpWin programme. The reproducibility of the results was guaranteed by measuring triplicate samples.

### 2.8. Photocatalytic activity determination

The photocatalytic activity was investigated with UV-Vis spectroscopy (Cary 50, Varian Inc., CA, USA), by bringing in contact the methylene blue (MB) with the photocatalytic active surface (zinc substrate covered by EP and EP with different GOs). The absorbance spectra of MB solutions were recorded after 1, 2, 24, 48, and 72 hours of exposition time. A calibration curve was plotted by measuring the absorbance of MB aqueous solutions with concentrations of 2 × 10^−5^, 10^−5^, 5 × 10^−6^, 4 × 10^−6^, 2.4 × 10^−6^, and 1.2 × 10^−6^ M at 665 nm, the maximum peak wavelength. To investigate the photocatalytic activity of the Zn/EP-GO systems and identify the specific components responsible for catalyzing the chemical reactions through light energy, we created multiple layers of EP doped with different functionalized GOs also on glass. Both glass and zinc samples had an active surface of 17.5 cm^2^ in 15 mL 10^−3^ M methylene blue aqueous solution.

The pre-treatment of the glass samples took place in several steps: first, the glass plates were washed with soapy water, then with 10% sulfuric acid, thoroughly rinsed with water, and dried. Then, they were wiped with cotton soaked in isopropanol, followed by the application of isopropanol onto them, and finally, the drying process.

In the preparation of the coated glass samples, we utilized the same dip-coating method to apply the layers, ensuring uniformity in parameters. This involved maintaining a concentration of 0.1 wt% of various GO nanoparticles in the EP resin, with a withdrawal speed of 5 cm min^−1^.

Several studies have been conducted to elucidate the MB degradation kinetics in an aqueous solution. In some reports^49–51^ the MB degradation rate has been calculated based on a pseudo-first kinetic model as it is described by the eqn (1):1
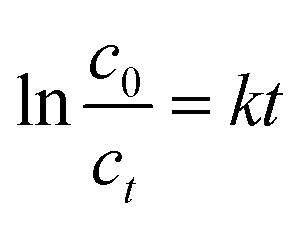


The *c*_0_ and *c*_*t*_ represent the MB initial concentration and after *t* time elapsed. The rate constant (*k*_obs_) can be determined as the plot of ln(*c*_0_/*c*_*t*_) *vs. t*. The MB solution concentration in a certain moment can be determined based on Lambert–Beer law (eqn (2)) from absorption spectra.

2 *A* = *ε*·*c*·*l*

The molar absorption coefficient *ε*, represents the slope of the calibration curve, that is the plot of maximum peak absorbance (*A*) determined from the spectra *vs.* concentration (*c*). In eqn (2)*l* represents the path length. The coatings efficiency is correlated with the amount of transformed MB, expressed by eqn (3):3
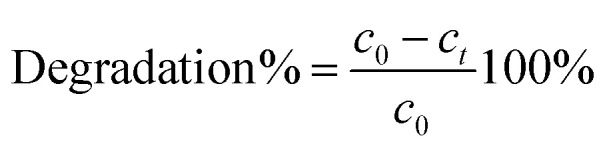


The symbols have the same meaning described above.

### 2.9. Coating adhesion

The adherence of the coatings was confirmed through the cross-hatch adhesion test employing an Elcometer Cross Hatch Adhesion Tester and categorized following the ASTM D3359 classification, specifically utilizing Method B (cross-cut).^48^ This test provides a visual indication of the adhesion quality by evaluating the extent of coating removal after making cuts through the coating layer. The adhesion was assessed for both dry coatings and coatings submerged in 10^−3^ M methylene blue aqueous solution for 3 days (the layers were dried 1 day before the adhesion test). Adhesion tests were made before and after photocatalysis to determine the changes in the adhesion properties due to photocatalysis. A 7 × 7 surface area was utilized, and the proportion of the region remaining after tape removal was determined.

### 2.10. Scanning electron microscopy measurements

A Hitachi SU8230 ultra-high-resolution scanning electron microscope was used to study the morphological and structural properties of the Zn/EP-different GO systems before and after the photocatalytic determination.

## 3 Results and discussion

### 3.1. Fourier transformed-infrared spectroscopy (FT-IR) measurements

To gain an understanding of potential bond formation between modified graphene oxide (GO) and epoxy resin (EP), FT-IR spectra were recorded. The FT-IR spectrums of the EP in the range of 2800–3000 cm^−1^ possess the bands attributed to the C–H stretching. The band at around 1300 cm^−1^ shows the C–O stretching and at 1000 cm^−1^ can be observed the vanishing peak of the epoxide ring (which is attributed to the cured epoxy).^52^

**Fig. 2:**
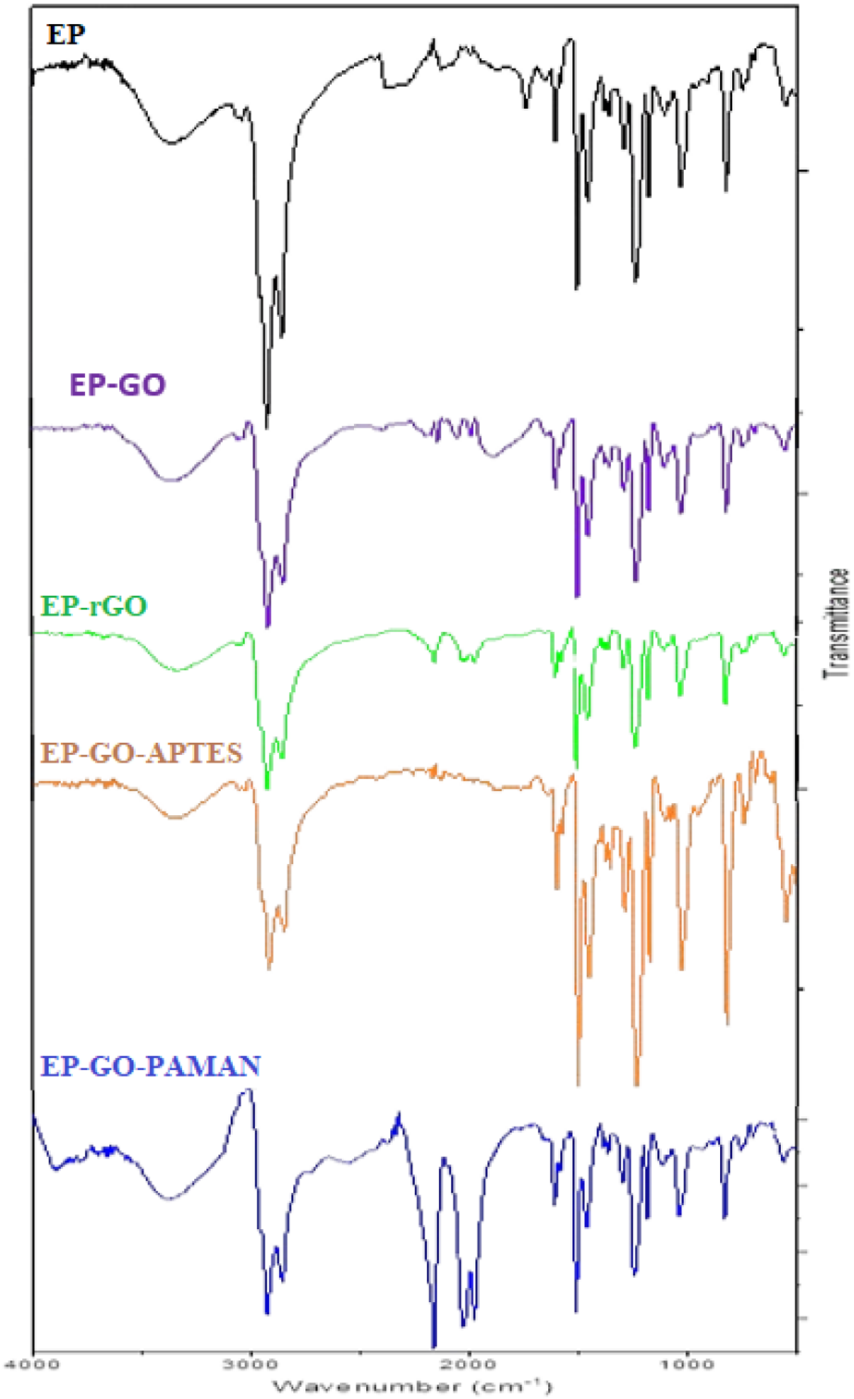
FT-IR spectra of neat and rGO, GO, GO-APTES, GO-PAMAM nanoparticles doped EP.

The representative signals of the GO appear in the case of EP-GO layers (Fig. 2). The spectra of EP-GO show vibration in the range of 3200–3550 cm^−1^ and 1400–1500 cm^−1^ which is related to the –OH groups and C=C aromatic bonds from bisphenol. Also, around 1700 cm^−1^ stretching vibration of C=O appears specific to the carboxyl groups. The bands attributed to the ether group (C–O–C) and alkoxy group (C–O) increased compared to the cured EP, as these groups can also be found on the surface of the GO.^7,53^ Since the rGO shares the same groups as the GO on its surface, one can observe the presence of similar bands, although they appear with reduced intensity due to less pronounced oxidation.

New bands become visible after GO-PAMAM incorporation in EP, proving the interactions between the nanoparticles and the resin. In the range of 3300–3400 cm^−1^ the stretching vibration of the N–H overlaps with the stretching of the O–H bonds.

In the case of GO-APTES embedment, the C–OH vibrations originating from hydroxy groups decrease in the EP samples. This observation substantiates that the functionalization process involving APTES and GO leads to a reaction where the ethoxysilane groups of APTES interact with the OH groups of GO, resulting in the release of ethanol and the formation of Si–O–C bonds.^7^ The Si–C peak appearing around 600 cm^−1^ suggests that there are Si–C bonds present in the material being analyzed. The superposition of the Si–O signal with the C–H bend around 900 cm^−1^ results in an increased peak in the spectrum.

### 3.2. Coating thickness

These measurements were performed to see whether the different type of functionalization influences the coating thickness. The obtained values are summarized in Table 1.

**Table 1:** Coating thickness of rGO, GO, GO-APTES, GO-PAMAM nanoparticles doped EP

Samples	Coating thickness/µm
Zn/EP	24.0
Zn/EP-GO	24.2
Zn/EP-rGO	23.6
Zn/EP-GO-APTES	26.5
Zn/EP-GO-PAMAM	28.0

The discernible conclusion is that, in cases where the filler contained amine groups, the doped coatings exhibited increased thickness. A possible explanation might involve the cross-linking of the epoxy (EP) matrix induced by the presence of these fillers. Amine groups, being nucleophilic, attack the electrophilic carbon of the epoxy group (oxirane ring) in the epoxy resin. This nucleophilic attack leads to the formation of a carbon–nitrogen bond. As the amine groups can form hydrogen bonds *via* their unpaired electrons with the glycidyl group of the epoxy resin, the exotherm curing reaction of the epoxy was accelerated, leading to a denser and better cross-linked polymer coating.^44^ The rate of curing can be adjusted by varying the structure and concentration of the amine catalyst.^54,55^ The larger volume of PAMAM dendrimer and its orientation inside the EP layer could explain the higher thickness of the coating. When the dendrimers are part of the EP layer, the increased volume allows for more deposition of the coating materials due to the increasing electrostatic interaction between the coating material and the surface. Proper orientation facilitates better alignment of dendrimer branches with the substrate, optimizing the electrostatic interactions and enhancing the adhesion of the coating.

### 3.3. Electrochemical measurements

The main goal of the electrochemical measurements was tracking the changes caused by the photocatalysis process on the anti-corrosion properties of the coatings. As the first step of electrochemical characterization, OCP measurements were performed. Table 2 presents the obtained values for the different samples after 30 minutes of immersion.

**Table 2:** Open circuit potential recorded after 30 minutes of immersion in 3 wt% NaCl solution of the epoxy coated samples

Samples	OCP (V *vs.* Ag/AgCl/KCl_sat_)
Zn	−0.995
Zn/EP	−0.967
Zn/EP-rGO	−0.988
Zn/EP-GO	−0.950
Zn/EP-GO-APTES	−0.923

It can be observed, that the epoxy-covered Zn substrate OCP value is higher, and this trend is amplified by GO and modified GO addition to the coating matrix. Generally, an increase in corrosion potential can be attributed to a decrease in the anodic reaction with the growth of a passive film. In our case, the shift to more positive potentials is due to the covering layers' property of blocking the electrolyte pass. In the presence of functionalized GO, the large number of oxygen-containing groups on the surface of GO can tremendously reduce the electrical conductivity and electrochemical activity. Therefore, GO can inhibit corrosion to a certain extent and ensure the corrosion resistance of the coating.^56^ Only the EP-rGO makes an exception from the previous statement, probably due to the increased agglomeration of this conductive material.

**Fig. 3:**
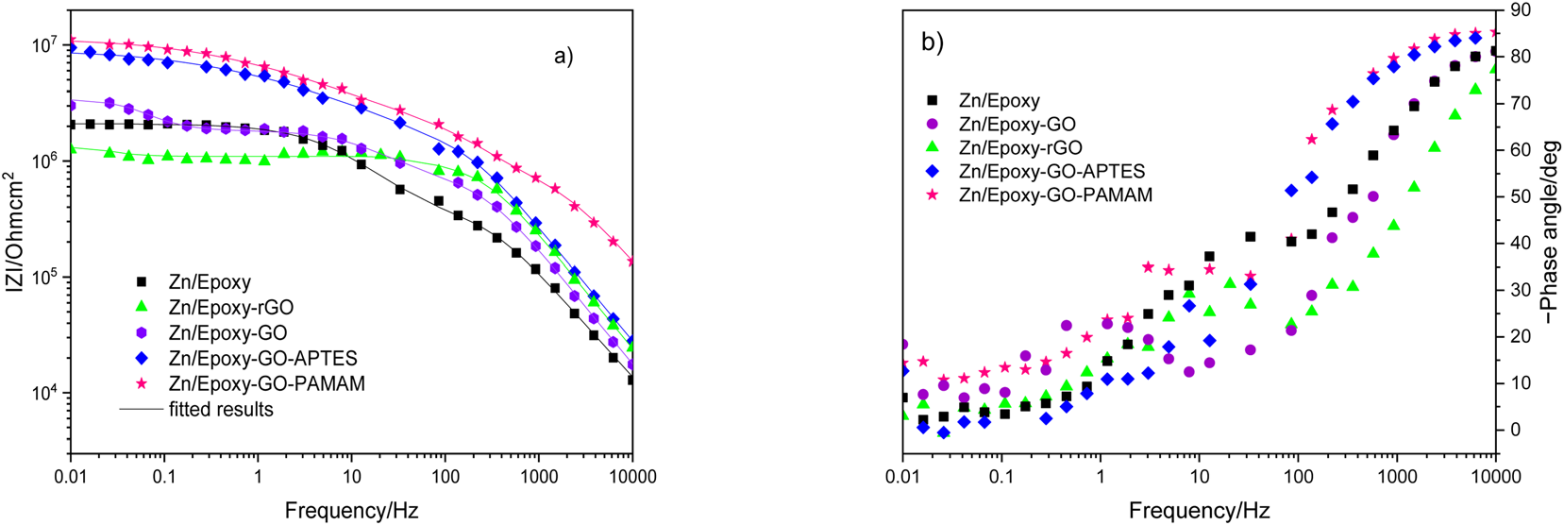
Bode magnitude (a) and phase angle (b) plots for the EP, EP-GO, EP-rGO, EP-GO-APTES, and EP-GO-PAMAM coated Zn substrates.

The samples were put through EIS measurements before (Fig. 3) and after they were soaked in MB solution and exposed illuminated, and the obtained Bode spectra of the studied systems were compared (Fig. 5). The barrier properties of the coatings are related to the values of the absolute impedance at 0.01 Hz ∣*Z*∣_0.01Hz_, the phase angle modulus at 10 kHz (*θ*_10kHz_), and the frequency determined at −45° phase angle so-called breakpoint frequency *f*_b_. Taking into account that ∣*Z*∣_0.01Hz_ values over 10^8^ Ω cm^2^ indicate excellent, while under 10^6^ Ω cm^2^ poor protection,^57^ one can declare that except the Zn/EP-rGO system all have good anti-corrosion performance (Fig. 3a). The highest impedance is obtained in the case of EP-GO-PAMAM, a possible explanation for this outcome is the GO-PAMAM particles possess the highest oxidation degree and the lowest electrical conductivity.^7^ Moreover, the high number of the amine groups from the PAMAM molecule could easily form hydrogen bonds with the glycidyl group in the EP component, resulting in a more compact and well-crosslinked polymer coating.^44^ Finally, the increased thickness of the coating is an additional factor that renders the EP-GO-PAMAM coating the most effective in corrosion protection. The rGO-EP coating's lower protective performance could be its electrical conductivity. rGO usually has higher electrical conductivity compared to GO due to the reduction process, which removes oxygen-containing functional groups, partially restoring the π-conjugated structure and increasing electrical conductivity. Higher electrical conductivity can lead to less effective electrochemical protection against corrosion processes. The phase angle value at high frequency, at 10 kHz (−*θ*_10kHz_),^58^ values vary from 0° – for uncoated metal – to 90° for a perfect undamaged coating, the lower values indicate diminished barrier properties, that permit electrolyte penetration through it. The −*θ*_10kHz_ are over 80° for the APTES and PAMAM-modified GO particles embedded epoxy coatings and around 80° for the neat and GO-modified epoxy (Fig. 3b). The lowest value was measured in the case of Zn/EP-rGO samples.

**Fig. 4:**
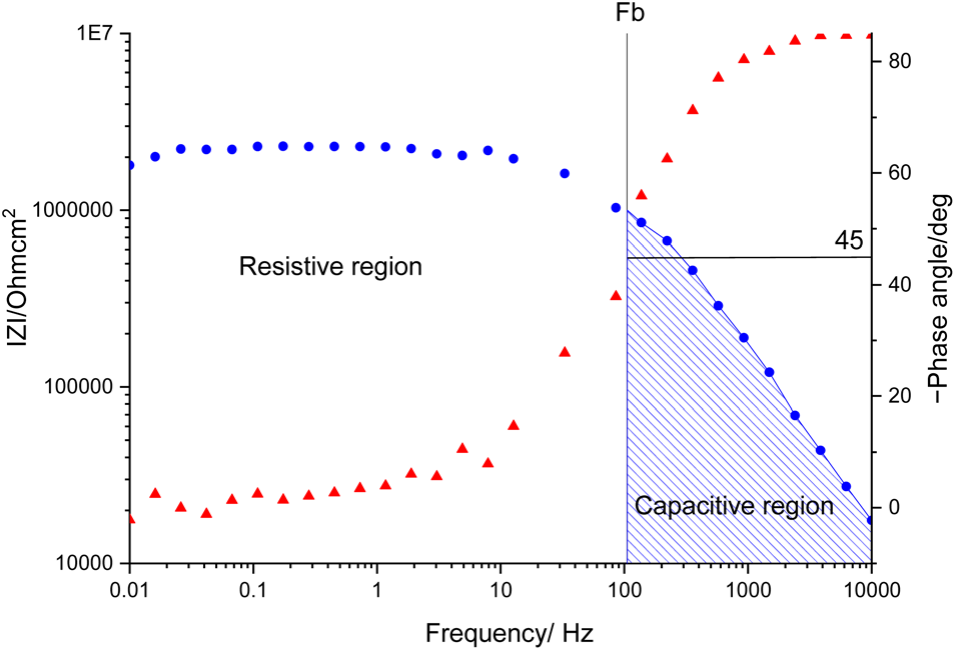
Bode diagram for a coated substrate.

The interpretation of the experimental spectra was conducted *via* both graphical analysis and equivalent circuit (EC) fitting. The above-mentioned characteristic features of the Bode plots depict the presence of a resistive region, under the horizontal line of ∣*Z*∣ and a capacitive domain (*A*_1_), the area beneath the slanted line (Fig. 4).

The coating damage resulting from wear, along with consequent delamination and adherence loss can be estimated (i) in wide frequency range analysis, based on the changes of resistive region/capacitive region areas and (ii) by single frequency analysis through variation of absolute impedance at 0.01 Hz (∣*Z*∣_0.01Hz_) or breakpoint frequency (*f*_b_) ∣*Z*∣_*f*_b__.^26^ The *D*_I_ delamination index described by eqn (4) indicates the decrease of the capacitive region area due to the photocatalysis induced delamination:4
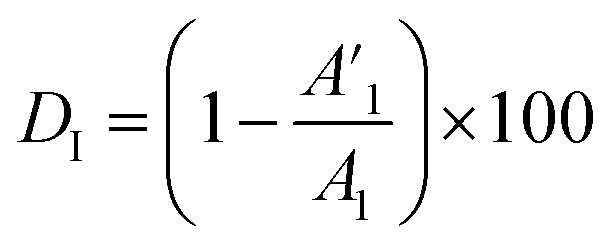
where *A*_1_ and 
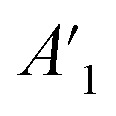
 stands for the capacitive region area before and after photocatalysis respectively. *D*_It_ delamination index expressed by eqn (5) represents the changes of the total area beneath the impedance modulus curve (before *A*_1t_ and 
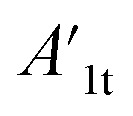
 after) due to the coating degradation:5
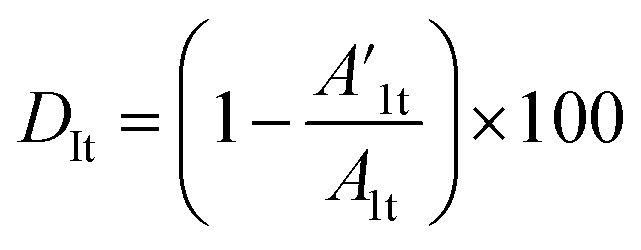
Finally, *D*_II_ represent the delamination index (eqn (6)) at a single frequency (*e.g.* 0.01 Hz or *f*_b_) and is defined with the absolute impedance values determined at specific frequency (∣*Z*∣_1_ and 
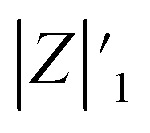
 before and after photocatalysis).6
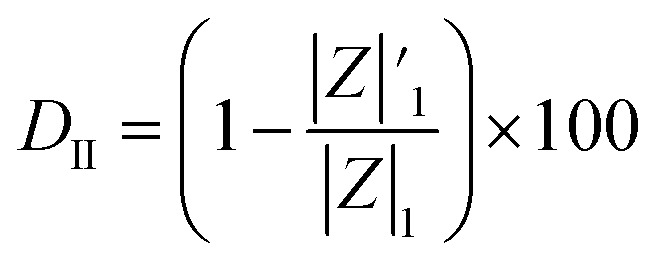


**Fig. 5:**
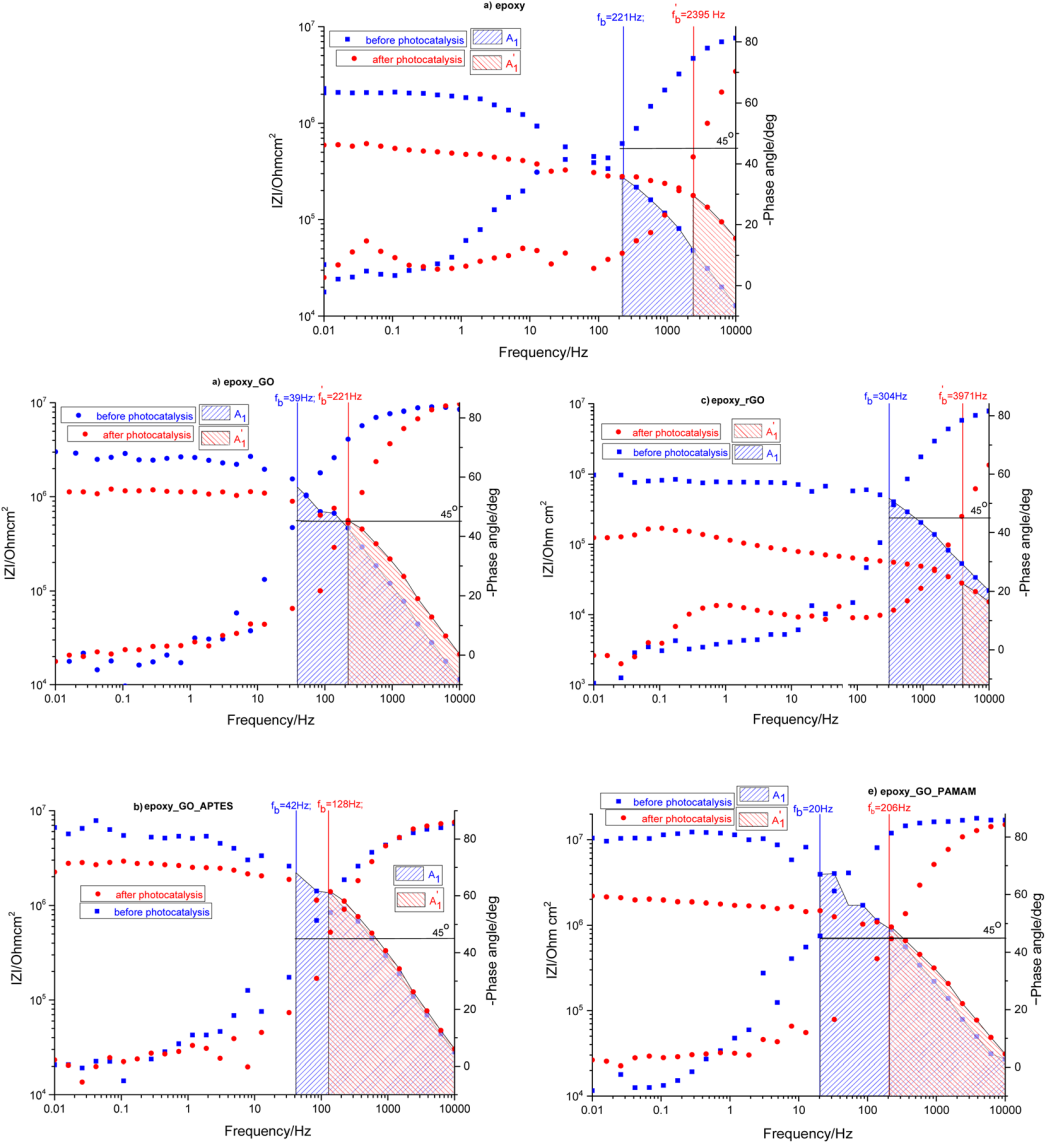
Comparison of Bode plots before and after photocatalysis for EP (a), EP-GO (b), EP-rGO (c), EP-GO-APTES (d) and EP-GO-PAMAM (e) coated Zn substrates.

The obtained Bode plots were compared and depicted in Fig. 5.

At first glance it can be observed that the magnitude of the impedance at low frequency, ∣*Z*∣_0.01Hz_, decreased in all cases, with an extent depending on epoxy matrix filler. Also, in all cases the resistive region, which is characteristic of the electrolyte penetration in the micropores of the coating, increased. The coating deterioration can also be seen in the narrowing of the capacitive regions (Fig. 5). The *f*_b_, correlated with the delaminated area, and absolute impedance values ∣*Z*∣_0.01Hz_ and ∣*Z*∣_*f*_b__ were determined and listed in Table 3 both before and after photocatalysis.

**Table 3:** Breakpoint frequencies (*f*_b_), the absolute impedance at 0.01 Hz (∣*Z*∣_0.01Hz_) and *f*_b_ (∣*Z*∣_*f*_b__) for the GO and modified-GO doped epoxy coatings on Zn substrate before and after photocatalysis based on graphical analysis

Sample	Before photocatalysis			After photocatalysis		
	*f*_b_/Hz	10^−5^·∣*Z*∣_*f*_b__/Ohmcm^2^	10^−5^·∣*Z*∣_0.01_/Ohmcm^2^	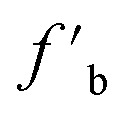 /Hz	10^−5^· 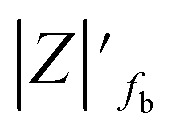 /Ohmcm^2^	10^−5^· 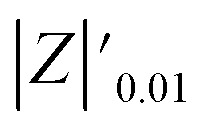 /Ohmcm^2^
Zn/EP	221	2.76	20.70	2395	1.78	5.93
Zn/EP-GO	39	12.66	30.20	221	5.62	11.29
Zn/EP-rGO	304	4.52	9.71	3971	0.29	1.24
Zn/EP-GO-APTES	42	22.06	66.24	128	13.86	22.43
Zn/EP-GO-PAMAM	20	39.30	104.63	206	9.62	21.90

The increased values of the *f*_b_ after photocatalysis in all cases indicate the coatings' delamination. For a quantitative comparison, coating delamination indexes *D*_I_ and *D*_II_ were calculated with eqn (4)–(6) and the obtained values are presented in Table 4. The detailed calculations can be found in the ESI Files Table S1.† The index *D*_I_ values from Table 4, represent the extent of capacitive region loss, while *D*_It_ indicates the degradation percentage of the coating in the whole frequency range after soaking.

**Table 4:** Delamination indexes *D*_I_ and *D*_II_ calculated for the GO and modified-GO doped epoxy coatings on Zn substrate before and after photocatalysis obtained from graphical analysis

	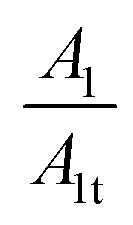	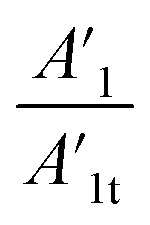	*D*_I_/%	*D*_It_/%	*D*_II_ at 0.01Hz/%
Zn/EP	0.09	0.06	87.87	82.41	71.35
Zn/EP-GO	0.40	0.43	64.19	66.67	62.61
Zn/EP-rGO	0.39	0.02	99.38	90.02	87.23
Zn/EP-GO-APTES	0.31	0.46	41.92	60.78	66.14
Zn/EP-GO-PAMAM	0.36	0.38	79.87	80.93	79.07

Also, in Table 4 the ratio 
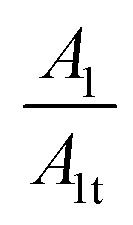
, 
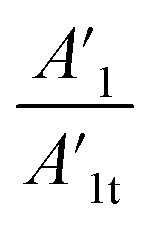
 representing the area of the capacitive region to the total area before and after photocatalysis are indicated.

In all cases, the total area beneath the impedance modulus curve decreased due to the photocatalysis. In the case of EP and rGO filled epoxy the capacitive area decreased, while in the case of GO-APTES can be observed exactly the opposite trend. In the case of GO and GO-PAMAM the capacitive area slightly increased, but it can be stated that photocatalysis does not change significantly the ratio of the two regions. This is in accordance with the *D*_I_ and *D*_It_ values, namely that in the case of the EP and rGO fillers mainly the delamination is the cause of coating damage and for GO-APTES the electrolyte penetration into the layer. In GO and GO-PAMAM cases, there is no essential difference between the two indicators, meaning that deterioration of the layer is caused by both effects roughly equally.

**Fig. 6:**
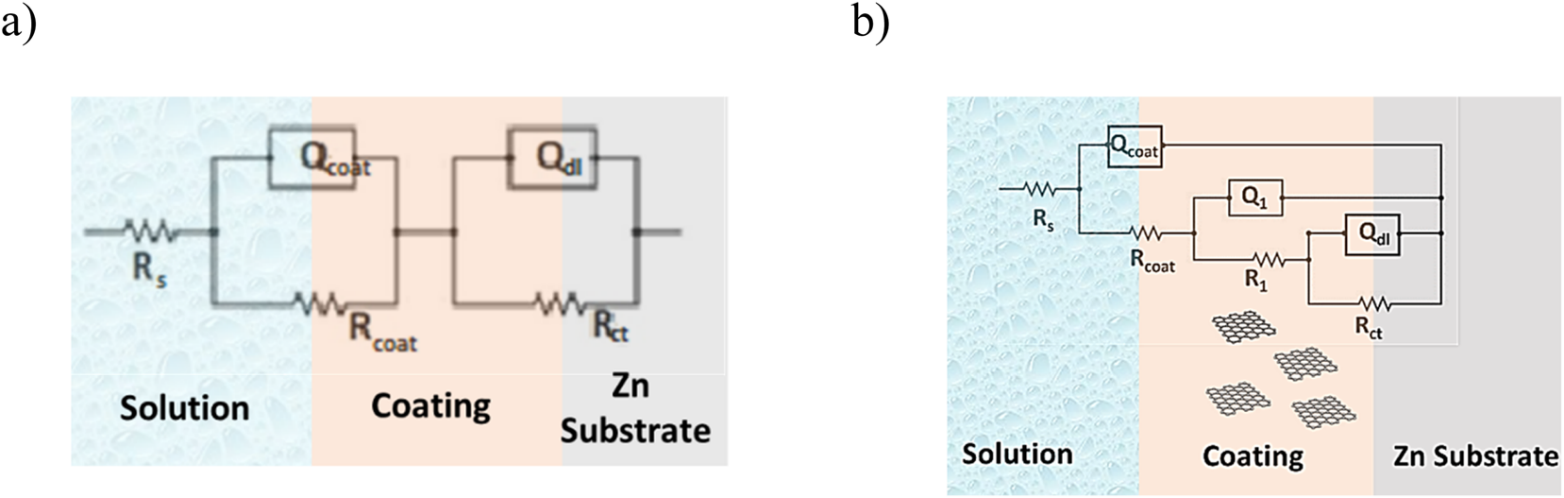
Equivalent circuit used at fitting EIS spectra for Zn/EP system (a) and the Zn/EP-various GO samples (b).

Another interpretation of the impedance spectra is through data modelling by fitting electrical equivalent circuits. For these measurements the fitted EC structure (Fig. 6) can be generally formulated by *R*_s_(*Q*_coat_(*R*_coat_(*Q*_1_(*R*_1_(*Q*_dl_*R*_ct_))))), although in some cases instead of constant phase element (CPE) is a capacitance (*C*). The detailed EC fitting can be found in the ESI File (Table S2 and S3).† CPE is used as a gauge of heterogeneity or roughness of the surface.^59^ In the above-mentioned formula, the *R*_s_, *R*_coat_, *R*_1_, *R*_ct_ represent the resistances, and *Q*_coat_, *Q*_1_, *Q*_dl_ the constant phase elements corresponding to solution, coating, filler, and charge transfer, respectively.

As can be observed in Fig. 6, the nanoparticles introduced in the coating matrix can represent an obstacle in the way of the diffusing species. The calculated parameters from the impedance data are presented in the ESI File, Table 2S† for spectra before photocatalysis and in Table 3S† for those recorded after. Considering that the polarization resistance (*R*_p_) (Table 5) is generated as a sum of the component resistances according to eqn (7)

7 *R*_p_ = *R*_coat_ + *R*_1_ + *R*_ct_

and it can be compared with the absolute impedance at 0.01 Hz. The delamination index (*D*_III_) is defined in a similar way to *D*_II_ for ∣*Z*∣_0.01_ by eqn (8) and represents the loss of the polarization resistance due to the interaction with the MB solution:8
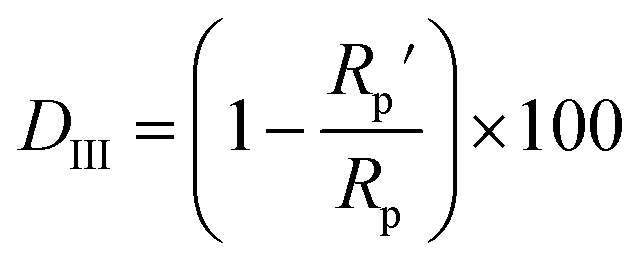
the values are inserted in Table 5. *R*_p_ and 
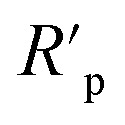
 represent the polarization resistance calculated from EC fitting to the spectra obtained before and after photocatalysis.

**Table 5:** Polarization resistance and delamination index *D*_III_ calculated for the GO and modified-GO doped epoxy coatings on Zn substrate before and after photocatalysis obtained from EC fitting

	EC fitting		
	Before photocatalysis	After photocatalysis	
Sample	*R*_p_/kΩ	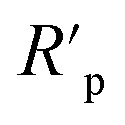 /kΩ	*D*_III_/%
Zn/EP	2103	788	62.53
Zn/EP-GO	3439	1152	66.50
Zn/EP-rGO	1517	124	91.82
Zn/EP-GO-APTES	9193	2771	69.85
Zn/EP-GO-PAMAM	11 606	2281	80.35

Comparing the *D*_II_ at 0.01 Hz (Table 4) and *D*_III_ values (Table 5), it can be seen that these are very close values, except for EP the difference is only a few percent between the two delamination indexes. Consequently, the conclusions that can be drawn from the equivalent circuit fitting complete those obtained from the graphical interpretation of the EIS data.

**Fig. 7:**
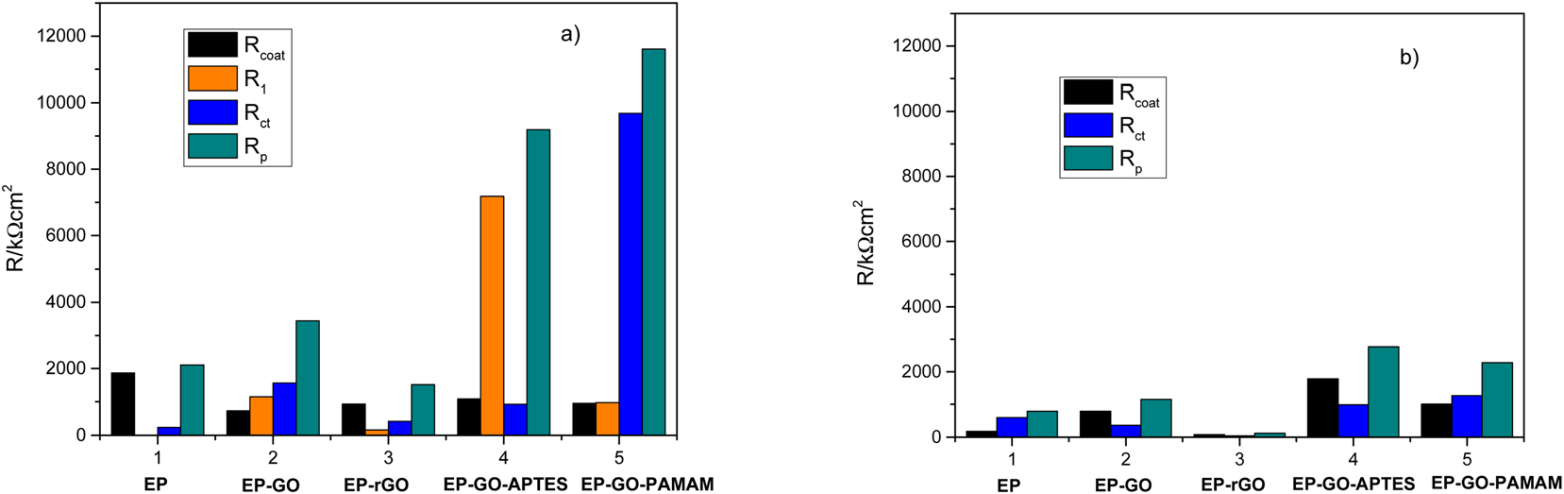
Resistive parameters of the equivalent circuits before (a) and after photocatalysis (b).

As expected, the GO-based fillers enhance the anti-corrosion performance of the epoxy coating due to the formation of a denser texture and better physical properties. It can be observed in Fig. 7a, that before photocatalysis the nanoparticles doped coatings resistance (*R*_coat_) due to the epoxy matrix is approximately the same in all cases. The effect of the introduction into the coating of the GO based nanofiller on resistance *R*_1_ value is influenced by its functionalization. The rGO nanoparticles degrade the overall effect of the composite while the GO-APTES and GO-PAMAM are increasing. The APTES functionalization of the GO reinforces its bonding to the EP providing insulating areas and, thus better barrier properties. The PAMAM functionalization of GO in the EP matrix can enhance the adhesion between the coating and the metal surface, thus hindering the electrolyte diffusion under the protective layer and consequently the corrosion process.^34^ This behaviour is reflected by the highest *R*_ct_, which represents the charge transfer resistance at the interface. Moreover, the EP-GO-PAMAM coatings exhibit the greatest thickness among the coatings. It is to be mentioned, that the PAMAM dendrimer can react with the functional groups of GO mostly on the edge, and the adhesion loss and electrolyte diffusion occur simultaneously, reflected both by *D*_I_, *D*_II_ indexes, and Fig. 7a and b histograms.

After photocatalysis (Fig. 7b) the coating failure can be observed in all cases. Although the diminution of polarization resistance is not negligible, based on their values, except EP-rGO, they can still be classified as good barrier coatings. The significant decrease of *R*_ct_ in the case of EP-GO-PAMAM coating can be explained by the loss of its adherence to the metal that allows the corrosion process to occur. From the comparison of the delamination indexes *D*_I_ and *D*_It_, it can be concluded that in both cases the lowest value was obtained for Zn/EP-GO-APTES samples, indicating the best anti-corrosion property based on a good coating adhesion and resistance against water permeation.

### 3.4. Photocatalytic activity determination

**Fig. 8:**
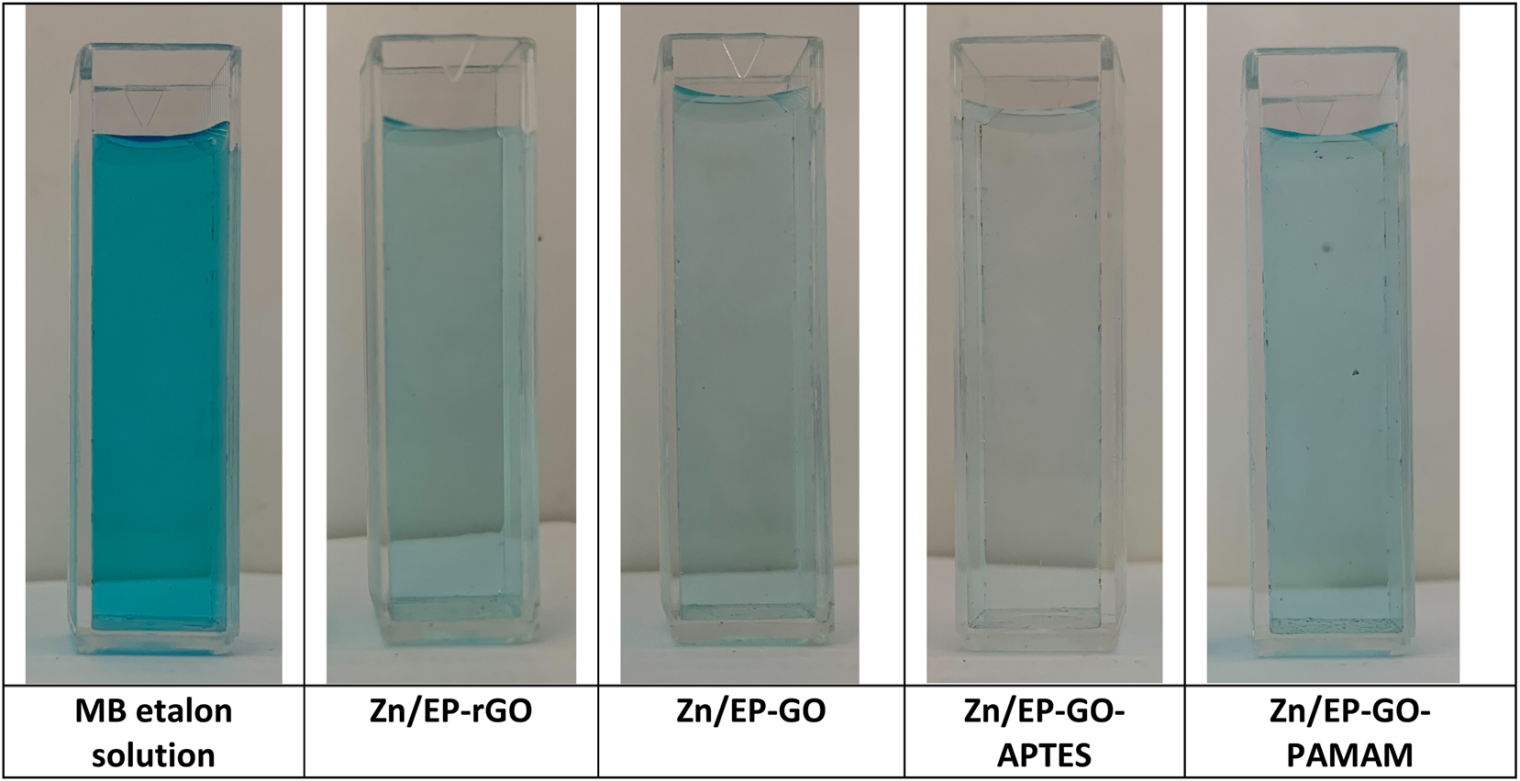
MB solution decomposition after 72 hours immersion of GO and modified GO nanoparticles doped EP coated Zn samples.

The photocatalytic performance of the EP, EP-GO, EP-rGO, EP-GO-APTES and EP-GO-PAMAM coatings on Zn wafers was determined toward time course decomposition of MB dye in visible light. The Zn/coatings samples were introduced in MB solution, and kept under visible light for well-determined period. The visual inspection of MB solution colours after 72 hours (Fig. 8) led to the conclusion that the GO-APTES nanoparticle-doped EP-coated Zn sample caused the greatest decrease in the dye's concentration.

**Fig. 9:**
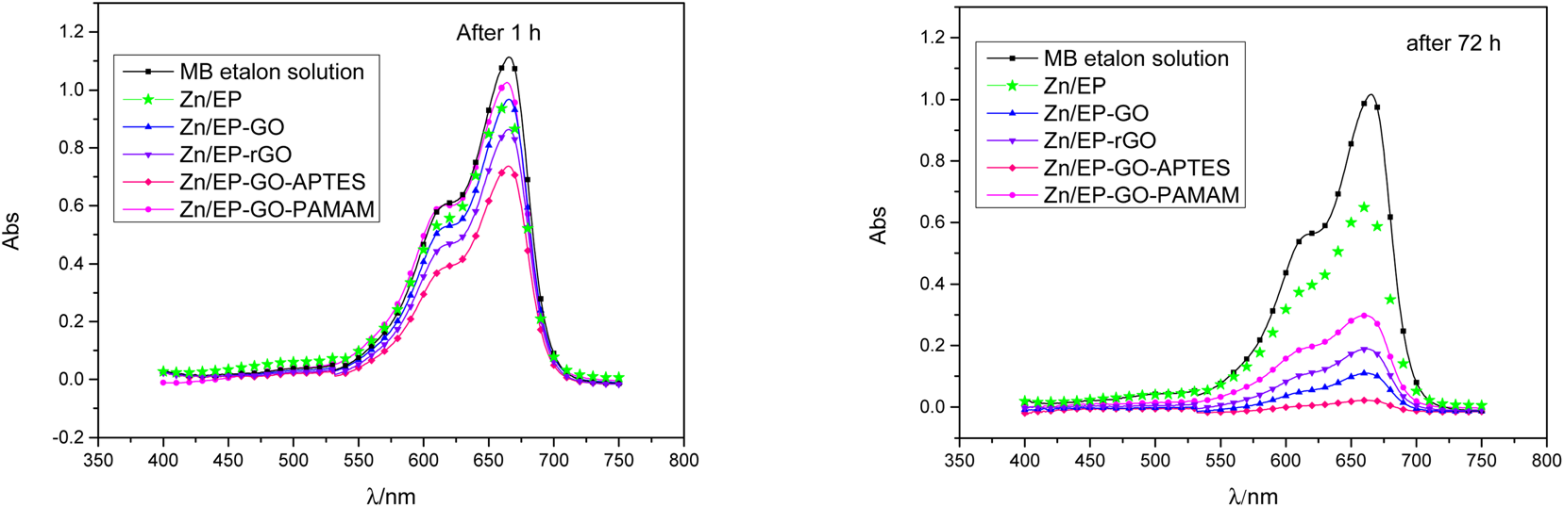
Decrease of the absorbance of MB dye solution due to the immersion for (a) 1 h and (b) 72 h of Zn/EP, Zn/EP-GO, Zn/EP-rGO, Zn/EP-GO-APTES and Zn/EP-GO-PAMAM samples.

For quantitative analysis absorbance measurements were performed in the Vis domain. The maximum peak absorbance – determined at approximately 665 nm – was monitored. The decrease in absorbance for the different samples are depicted in Fig. 9 after 1 and 72 hours exposition. It was observed that the epoxy coatings doped with modified GO nanoparticles caused varying degrees of MB decomposition, resulting in a different decrease in absorbance maximum. The least effective in the dye degradation was Zn/EP-GO-PAMAM sample. It can be observed that, after the same time elapsed, in both cases, the Zn/EP-GO-APTES sample caused the largest decrease in absorbance. Moreover, after 72 hours in this case is noticeable the maximum discoloration (Fig. 8) due to the advanced MB dye decomposition and a barely noticeable maximum peak on the absorbance spectrum (Fig. 9).

**Fig. 10:**
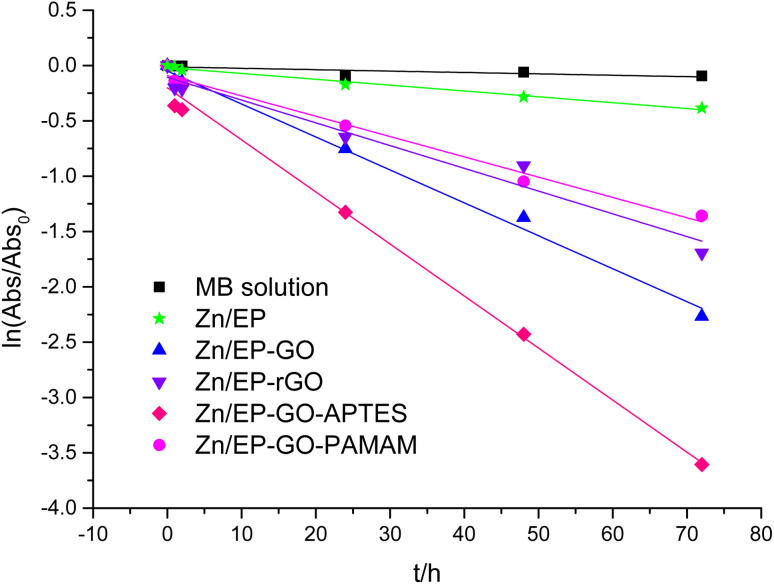
Pseudo-first order kinetic model for the MB dye degradation due to the immersion of Zn/EP, Zn/EP-GO, Zn/EP-rGO, Zn/EP-GO-APTES, and Zn/EP-GO-PAMAM samples.

For etalon MB solution calibration curve was plotted. Based on the Lambert–Beer law (eqn (2)) MB concentrations can be determined for different photocatalysis periods. Taking into account, the relationship between the MB solution concentrations and absorbance, in eqn (1) instead of *c*_o_/*c*_*t*_ the Abs_o_/Abs_*t*_ ratio can be used. The degradation reaction rate constant (*k*_obs_), was calculated according to eqn (1) as the slopes of the curves in Fig. 10, and inserted in Table 6. Also, the MB degradation% (eqn (3)) was determined (Table 6).

**Table 6:** MB degradation reaction rate constant due to the immersion of Zn/EP, Zn/EP-GO, Zn/EP-rGO, Zn/EP-GO-APTES and Zn/EP-GO-PAMAM samples

Sample	*k*_obs_/h	*R* ^2^	MB degradation (%)
Zn/EP	0.0053	0.9824	31.83
Zn/EP-GO	0.0390	0.9707	95.47
Zn/EP-rGO	0.0276	0.9644	88.65
Zn/EP-GO-APTES	0.0759	0.9876	99.95
Zn/EP-GO-PAMAM	0.0208	0.9898	79.23

The decomposition rate of the MB self-degrading dye is low, and the degradation% of the solution is between 7–10%, depending on the conditions, the process is accelerated only in the presence of a catalyst. In order to conclude the role of the Zn substrate, the same coatings were also applied to a glass substrate and subjected to similar tests as those formed on metal. The obtained values for MB degradation% due to the effect of different EP and GO-based nanofillers modified EP coatings on glass substrates are very similar to those obtained for the non-catalysed tests, as are presented in ESI File, Table S4 and Fig. S1–S3.† So, it can be concluded that the presence of Zn and ZnO layers naturally existing on the Zn substrate is critical for the reaction to take place and that the GO nanofillers doped layers alone are inadequate for this purpose. The increased number of functional groups on GO-APTES nanofiller provides good bonding possibilities both to the substrate and epoxy matrix. The addition of silane-functionalized GO in EP coatings enhances adhesion to the metal surface, forming covalent Si–O-metal bonds and increasing linkage density between the coating and the substrate,^44^ evidence supporting this observation is presented in Section 3.5 titled “Coating Adhesion”. Though GO-PAMAM also has several functional groups, because these are situated on the edge only and are large, they rather hinder than help the transformation in comparison with GO-APTES.

The best photocatalytic effect was provided by the Zn/EP-GO-APTES sample with a 99.95% degradation of the MB. Also, in this case, the polarization resistance decreases due to the soaking in the MB solution.

### 3.5. Coating adhesion

**Fig. 11:**
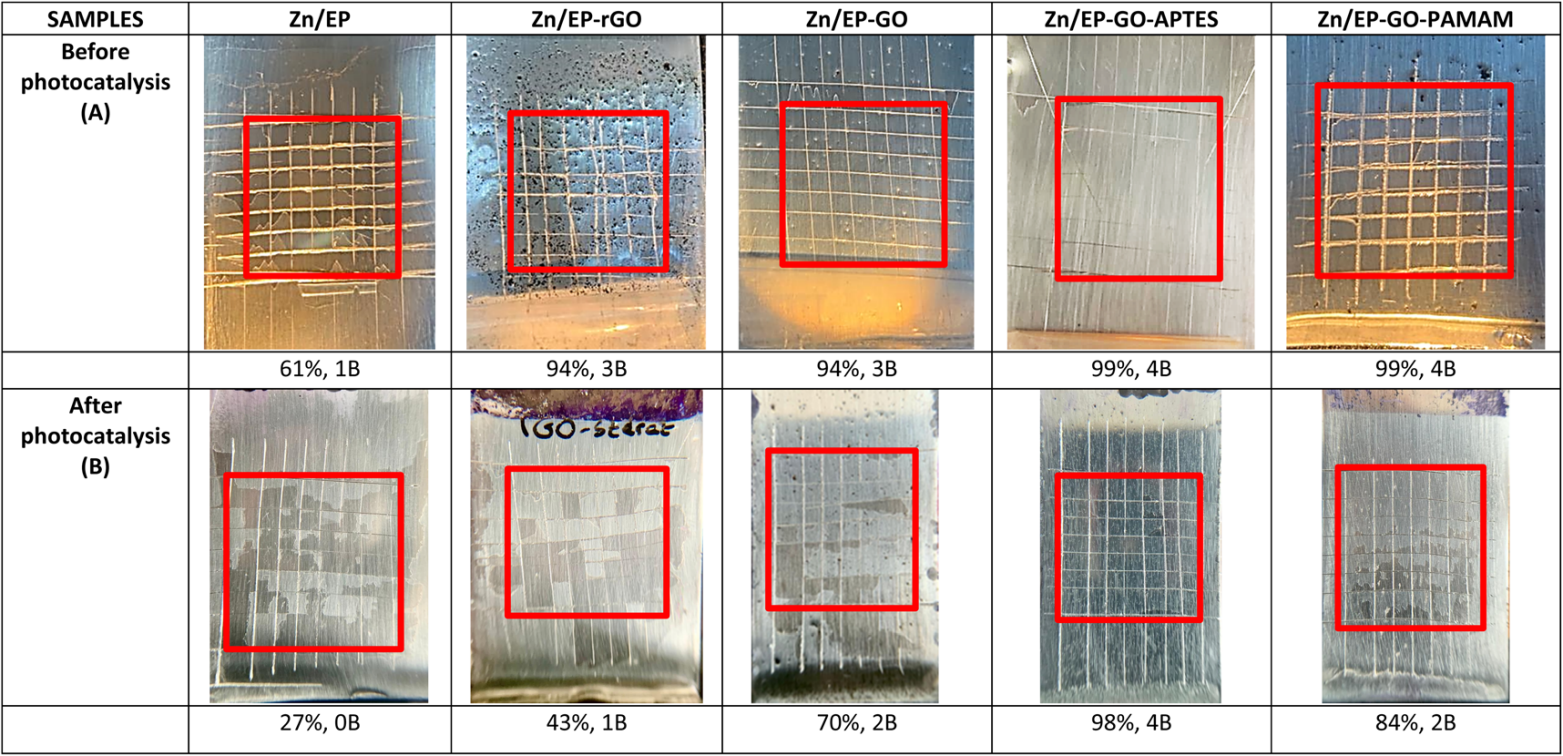
Cross-hatch adhesion test results for coated samples (A) before photocatalysis; (B) after photocatalysis. The percentages show the adhesion of the coatings, followed by the ASTM D3359 Classification.

The cross-hatch adhesion test results (Fig. 11) demonstrated that the presence of amine groups on the surface of the GO nanofillers (GO modified with APTES and PAMAM dendrimer), which were incorporated into EP matrix, greatly improved the adhesion of the systems and also maintained good bonding even after photocatalysis.

The adhesion loss can be calculated by using eqn (9) and the results are presented in Table 7. It can be concluded that Zn/EP-GO-APTES system exhibits minimal loss, maintaining nearly its initial adhesion after photocatalysis. The incorporation of silane-functionalized GO nanosheets into epoxy coatings enhances adhesion to the metal surface. Organosilanes serve as effective adhesion promoters, and the utilization of silane-modified GO in epoxy coatings results in the establishment of covalent Si–O-metal bonds between the silane and the metal surface hydroxyls. This process increases the density of linkages between the coating and the substrate.^44^ On the other hand, the EP with embedded PAMAM-modified GO shows an adhesion loss of 15.15%. In both cases, the smaller adhesion loss can be attributed to the presence of amines, which contribute to increased cross-linking in the composite, thereby enhancing its barrier properties. Alternatively, the interaction of amines with the metal oxide found on the substrate's surface may lead to improved adhesion too. In the case of EP-GO-PAMAM the increased adhesion loss in comparison with the EP-GO-APTES can be due to the positions of the amino-groups, that they are situated only on the edges.9
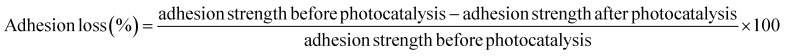


**Table 7:** Adhesion loss of the EP coated samples after 3 days of continuous exposure to MB solution

Samples	Zn/EP	Zn/EP-rGO	Zn/EP-GO	Zn/EP-GO-APTES	Zn/EP-GO-PAMAM
Adhesion loss (%)	55.74	54.26	25.53	1.02	15.15

These results are in good agreement with values obtained from electrochemical measurements, namely the extent of delamination reflected by *D*_I_ values and the highest obtained for EP-rGO and the lowest for EP-GO-APTES.

### 3.6. Scanning electron microscopy measurements

**Fig. 12:**
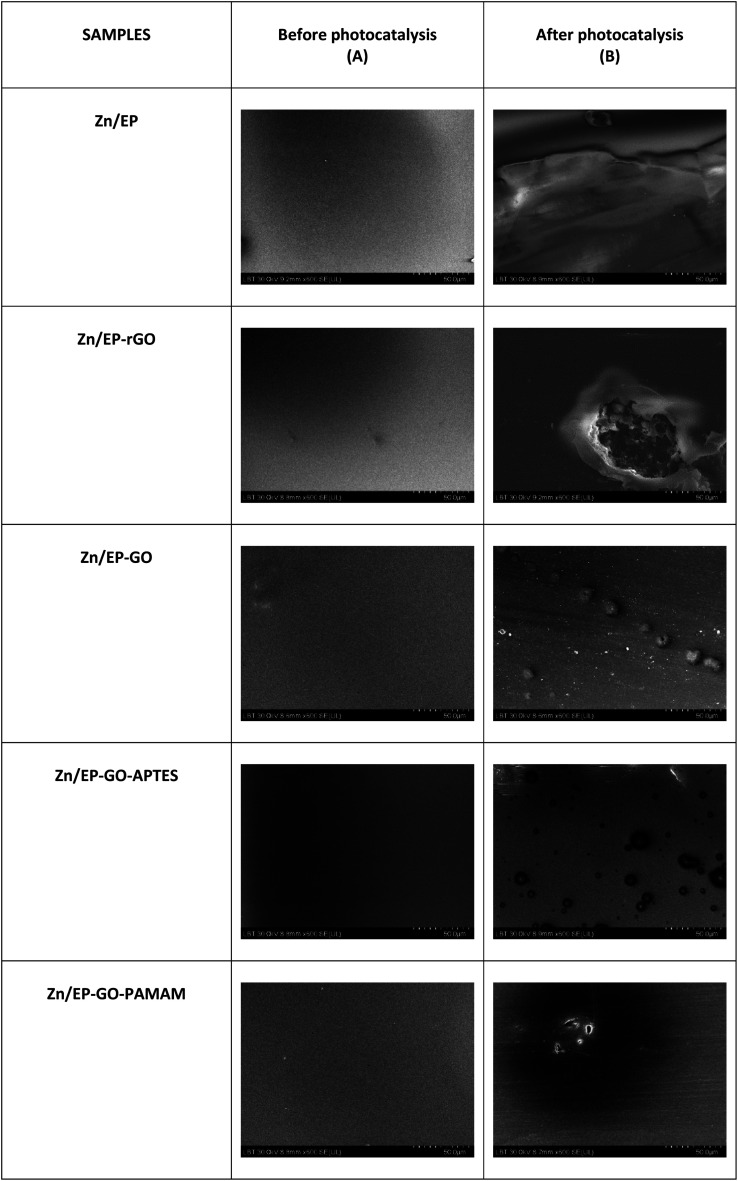
SEM images of coated samples (A) before photocatalysis; (B) after photocatalysis.

After 3 days of immersion in harmful MB solution, SEM images show that the EP had the greatest solvent-induced swelling, confirmed also by its poorest adhesion. It can be seen that EP-rGO forms agglomerates and accelerates corrosion. The rGO-EP coating's lower protective performance, as also evidenced by electrochemical measurements, can be attributed to this uneven distribution that can create discontinuities or weak points in the coating, reducing its protective effectiveness. The same agglomerate formation can be observed also for EP-GO-PAMAM. In these two cases the photo-degradation effect is decreased, compared to the Zn/EP-GO-APTES, where we have a more dispersed surface and the highest degradation of MB dye (Fig. 12).

## 4 Conclusions

Thin epoxy (EP) layers embedding 0.1 wt% graphene oxide (GO), reduced graphene oxide (rGO), and modified graphene oxide with (3-aminopropyl)-triethoxysilane (APTES) or poly(amidoamine) (PAMAM) dendrimer were applied on a zinc (Zn) substrate using the dip-coating method.

Correlations between corrosion resistance and photocatalytic properties of the coatings (effect of exposure to MB and light) can be summarized as follows: before exposure, the best corrosion resistance is exhibited by Zn/EP-GO-PAMAM samples, which could be explained by their very good adhesion, due to the presence of amino groups and to its highest thickness. Furthermore, the GO-PAMAM nanosheets possess the highest oxidation degree and the lowest electrical conductivity. The degradation of the coating by exposure to MB solution takes place with the smallest reaction rate constant (*k*_obs_), but the delamination of the coating leads finally to an important loss of adhesion, and, consequently to a smaller corrosion resistance.

Low delamination despite of the high photocatalytic activity was noticed for the Zn/EP-GO-APTES sample, at an acceptable corrosion resistance. By calculating the breakpoint frequency from EIS spectra, it was proved that the protective properties loss is due to coatings delamination during exposure to MB solution, the EP-GO-APTES still retaining the best adhesion of the coating, 98% remaining on Zn after cross-hatch test, because of the silane-functionalized GO in EP coatings, which enhances adhesion to the metal surface, forming covalent Si–O-metal bonds and increasing linkage density between the coating and the substrate.

All things considered, the Zn/EP-GO-APTES system shows the best ability to break down organic pollutants, keeping a good anti-corrosive property and adhesion. These features make EP-GO-APTES a preferred candidate for anti-corrosion protection coating of Zn substrate with a promising ability to degrade organic pollutants.

## Author contributions

Conceptualization, T.-R. O., G. S. Sz and L. M. M. methodology T.-R. O., G. S. Sz, G. K.; investigation, T.-R. O., B. T.; writing – original draft preparation, T.-R. O., G. S. Sz, writing – review and editing, G. S. Sz., L. M. M. and G. K. All authors have read and agreed to the published version of the manuscript.

## Conflicts of interest

There are no conflicts to declare.

## Supplementary Material

RA-014-D4RA00413B-s001
