# Supplementary material for: Correlations between the anti-corrosion properties and the photocatalytic behavior of epoxy coatings incorporating modified graphene oxide deposited on a zinc substrate
Source: RSC Adv. 2024 Apr 3;14(16):10826–41. doi: 10.1039/d4ra00413b (PMC10989242; doi:10.1039/d4ra00413b)
Supplement: RA-014-D4RA00413B-s001 [file RA-014-D4RA00413B-s001.pdf]

Electronic Supplementary Material (ESI) for RSC Advances.
This journal is © The Royal Society of Chemistry 2024

Supplementary Material

Correlations between the Anti-Corrosion Properties and the Photocatalytic Behavior of Epoxy Coatings Incorporating Modified Graphene Oxide Deposited on Zinc Substrate

Tamara-Rita Ovari[1], Boglárka Trufán[1], Gabriel Katona [2], Gabriella Szabó[2,*]

and Liana Maria Muresan [1]

Table S1. Bode plots parameters obtained by graphical analysis of the spectra Figure 5

Sample	10^{-9}* A_1	10^{-9}* A_1'	$\frac{A_1}{A_{1t}}$	10^{-9}* A_{1t}	10^{-9}* A_{1t}'	$\frac{A_1'}{A_{1t}'}$	BPhc 10^{-9}x (A_{1t}- A_1)	BPhc%	APhc 10^{-9}x (A_{1t}'- A_1')	APhc%
Zn/EP	0.99	0.12	0.09	11.03	1.94	0.06	10.04	91.02	1.82	93.81
Zn/EP-GO	5.81	2.08	0.40	14.52	4.84	0.43	8.71	59.98	2.76	57.02
Zn/EP-rGO	1.61	0.01	0.39	4.11	0.41	0.02	2.50	60.83	0.40	97.56
Zn/EP-APTES-GO	9.85	5.72	0.31	31.85	12.49	0.46	22.00	69.07	6.77	54.20
Zn/EP-PAMAM-GO	18.63	3.75	0.36	51.14	9.75	0.38	32.51	63.57	6.00	61.53

The meaning of the parameters in the Table 1S are:

- A_1, A_1' capacitive region area before and after photocatalysis
- A_{1t}, A_{1t}' total area beneath the impedance modulus curve before and after photocatalysis.
- $\frac{A_1}{A_{1t}}$, $\frac{A_1'}{A_{1t}'}$ the ratio of the area of the capacitive region to the total area before and after photocatalysis.
- $BPhc$ (A_{1t}- A_1) resistive region area before photocatalysis,
- APhc (A_{1t}'- A_1') resistive region area after photocatalysis
- $BPhc$ % resistive region % before photocatalysis

- ➢ APhc % resistive region % after photocatalysis

Table S2 Equivalent circuits and EC parameters for the recorded EIS spectra before photocatalysis

**Sample**		**R_s (kΩ cm^2)**	**Q_{coat} (μSs^n/cm^2)**	**n**	**R_{coat} (kΩ cm^2)**	**Q_1 (μSs^n/cm^2)**	**n**	**R_1 (kΩ cm^2)**	**Q_{dl} (μSs^n/cm^2)**	**n**	**R_{ct} (kΩ cm^2)**	**R_p = R_{coat}+R_{ct} (+R_1) (kΩ cm^2)**	**Chi^2**
Zn/EP	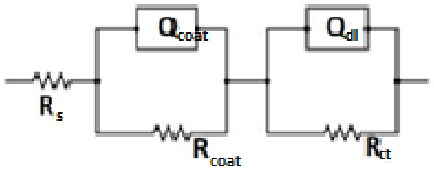	~0	3.4×10^{-2}	0.8	1867	-	-	-	2.7×10^{-3}	0.9	236	2103	4.31×10^{-3}
Zn/EP-rGO	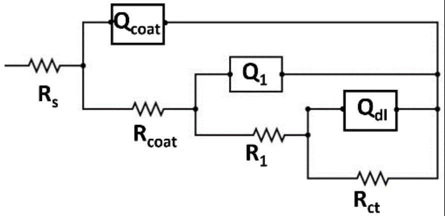	2.63	7.04×10^{-4}	0.9	930	1.51×10^{-2}	1	162	39.42	1	425	1517	8.47×10^{-3}
Zn/EP-GO	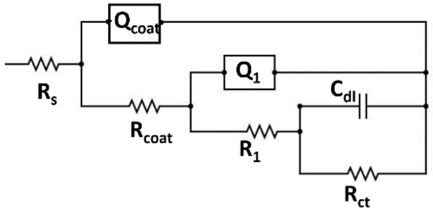	1.43	9.67×10^{-4}	0.9	728	2.40×10^{-2}	0.8	1146	1.98		1565	3439	6.57×10^{-3}
Zn/EP-GO-APTES	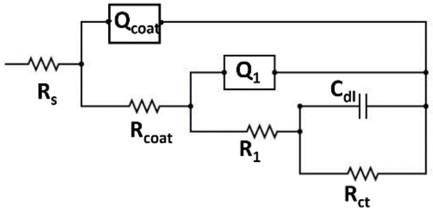	2.40	2.54×10^{-3}	0.9	1083	6.76×10^{-2}	0.5	7184	9.31×10^{-4}		923	9193	6.97×10^{-3}

Zn/EP-GO-PAMAM	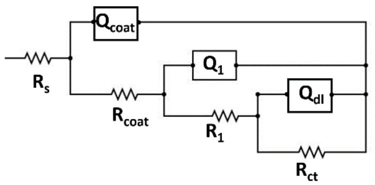	11.02	2.38×10^{-4}	0.9	950	5.34×10^{-4}	1	969	4.77×10^{-3}	0.5	9687	11606	3.43×10^{-3}

Table S3 Equivalent circuits and EC parameters for the recorded EIS spectra after photocatalysis

**Sample**		**R_s (kΩ cm^2)**	**Q_{coat} (μSs^n/cm^2)**	**n**	**R_{coat} (kΩ cm^2)**	**Q_{dl} (μSs^n/cm^2)**	**n**	**R_{ct} (kΩ cm^2)**	**$R_p = R_{coat}+R_{ct}$ (kΩ cm^2)**	**Chi^2**
Zn/EP	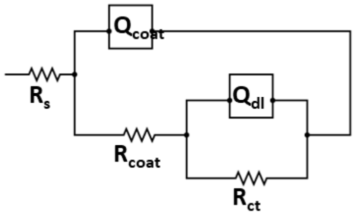	3.72	2.36×10^{-4}	0.9	182	1.19	0.3	606	788	5.48×10^{-3}
Zn/EP-GO	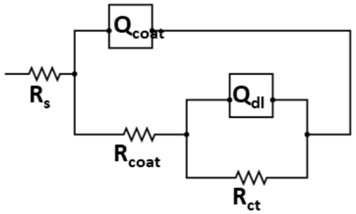	1.24	8.99×10^{-4}	0.9	783	7.83×10^{-2}	0.9	369	1152	4.92×10^{-3}
Zn/EP-rGO	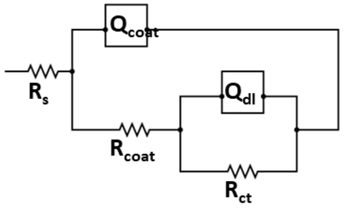	0.14	1.33	0.6	81	342	0.8	43	124	8.45×10^{-4}
Zn/EP-GO-APTES	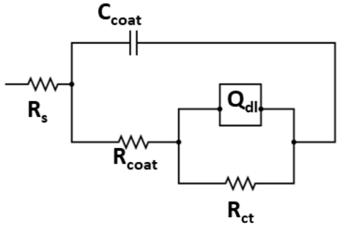	2.37	5.26×10^{-4}		1783	1.06×10^{-1}	0.6	988	2771	4.54×10^{-3}

Zn/EP-GO-PAMAM	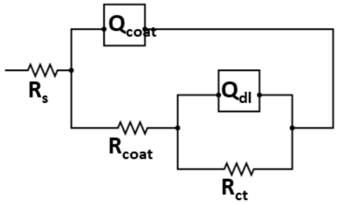	2.47	5.70×10^{-4}	0.9	1012	3.15×10^{-1}	0.3	1269	2281	2.79×10^{-3}

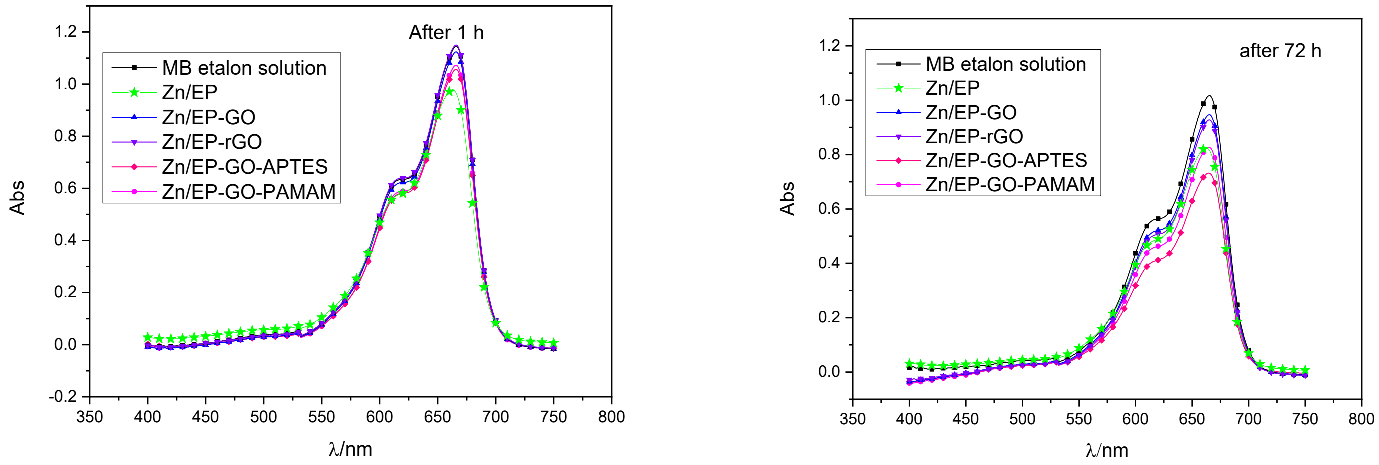

Figure S1. Decrease of the absorbance of MB dye solution due to the immersion for a) 1 h and b) 72h of Zn/EP, Zn/EP-GO, Zn/EP-rGO, Zn/EP-GO-APTES and Zn/EP-GO-PAMAM samples kept in dark for reference.

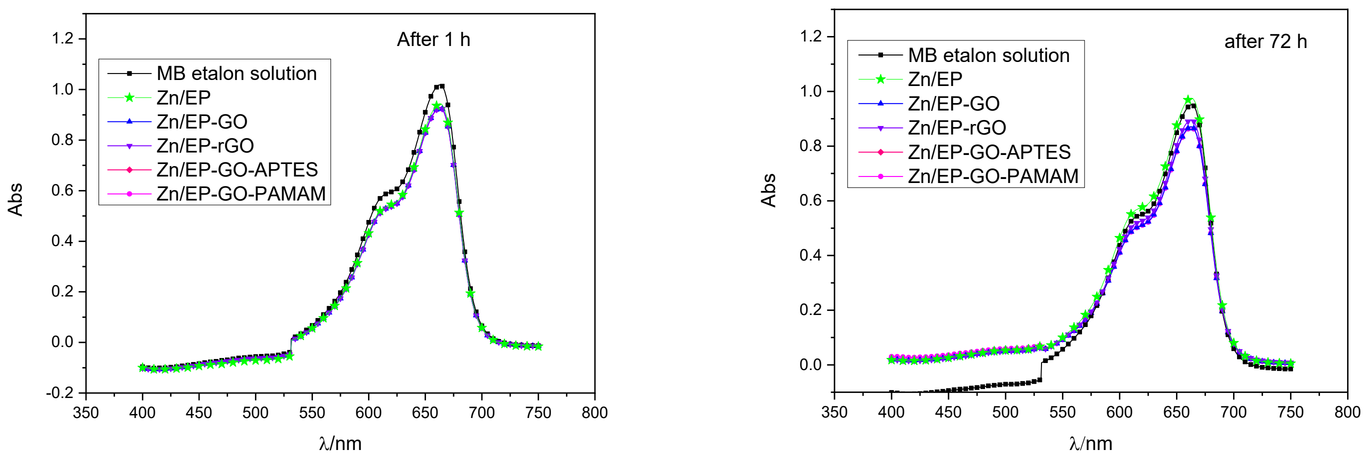

Figure S2. Decrease of the absorbance of MB dye solution due to the immersion for a) 1 h and b) 72h of EP, EP-GO, EP-rGO, EP-GO-APTES and EP-GO-PAMAM coatings on glass substrate.

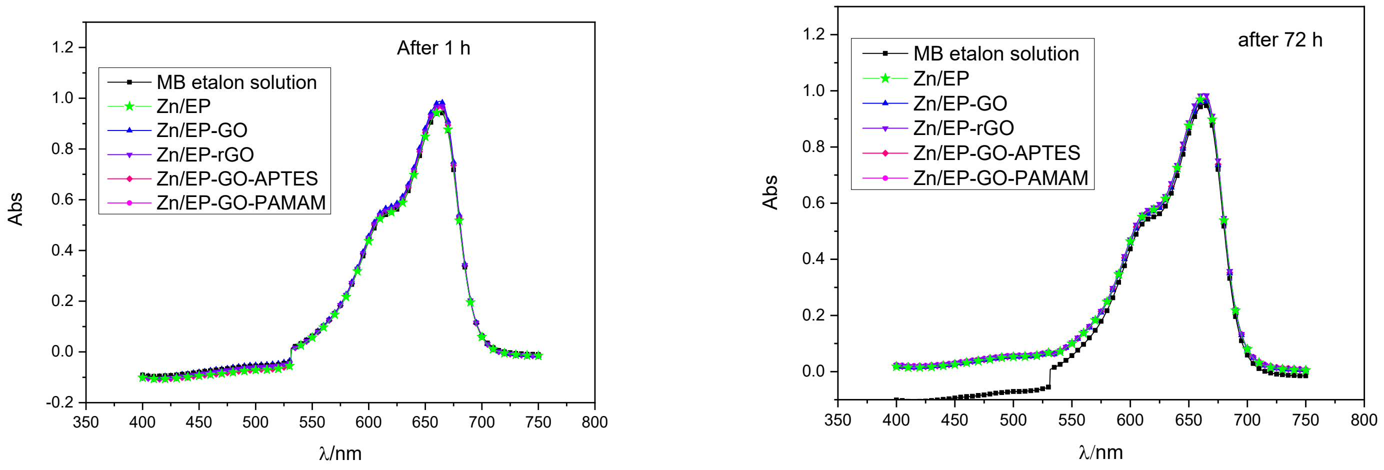

Figure S3. Decrease of the absorbance of MB dye solution due to the immersion for a) 1 h and b) 72h of EP, EP-GO, EP-rGO, EP-GO-APTES and EP-GO-PAMAM coatings on glass substrate kept in dark for reference.

Table S4. Photodegradation of MB on glass substrates, coated with EP resin and EP containing different GOs

**Coatings**	**MB degradation % Light**	**MB degradation % Dark**
	**Glass Substrate**	**Glass Substrate**
MB etalon solution	9.9	7.8
EP	7.7	5.8
EP-GO	14.1	5.5
EP-rGO	14.1	5.5
EP-GO-APTES	12.5	4.0
EP-GO-PAMAM	7.9	5.0
